# Supplementary material for: Elastic porous microspheres/extracellular matrix hydrogel injectable composites releasing dual bio-factors enable tissue regeneration
Source: Nat Commun. 2024 Feb 14;15:1377. doi: 10.1038/s41467-024-45764-4 (PMC10866888; doi:10.1038/s41467-024-45764-4)
Supplement: Supplementary file 1 — Supplementary Information [file 41467_2024_45764_MOESM1_ESM.pdf]

**Supplementary information for**

Elastic porous microspheres/extracellular matrix hydrogel injectable composites releasing dual bio-factors enable tissue regeneration

**Authors list:** Yi Li[1#], Siyang Liu[1#], Jingjing Zhang[2#], Yumeng Wang[1], Hongjiang Lu[1], Yuexi Zhang[3], Guangzhou Song[1], Fanhua Niu[1], Yufan Shen[1], Adam C. Midgley[1], Wen Li[1*], Deling Kong[1*], Meifeng Zhu[1*]

**Affiliations:** [1]College of Life Sciences, Key Laboratory of Bioactive Materials (Ministry of Education), State Key Laboratory of Medicinal Chemical Biology, Nankai University, Tianjin 300071, China.

[2] Chifeng Municipal Hospital, Chifeng, Inner Mongolia 024000, China

[3] The Third Affiliated Hospital of Wenzhou Medical University, Wenzhou, Zhejiang 325200, P. R. China

# These authors contributed equally to this work

*To whom the correspondence should be addressed: Wen Li, e-mail: liwen1182613400@163.com; Deling Kong, e-mail: kongdeling@nankai.edu.cn; Meifeng Zhu, e-mail: zhumeifeng@nankai.edu.cn;

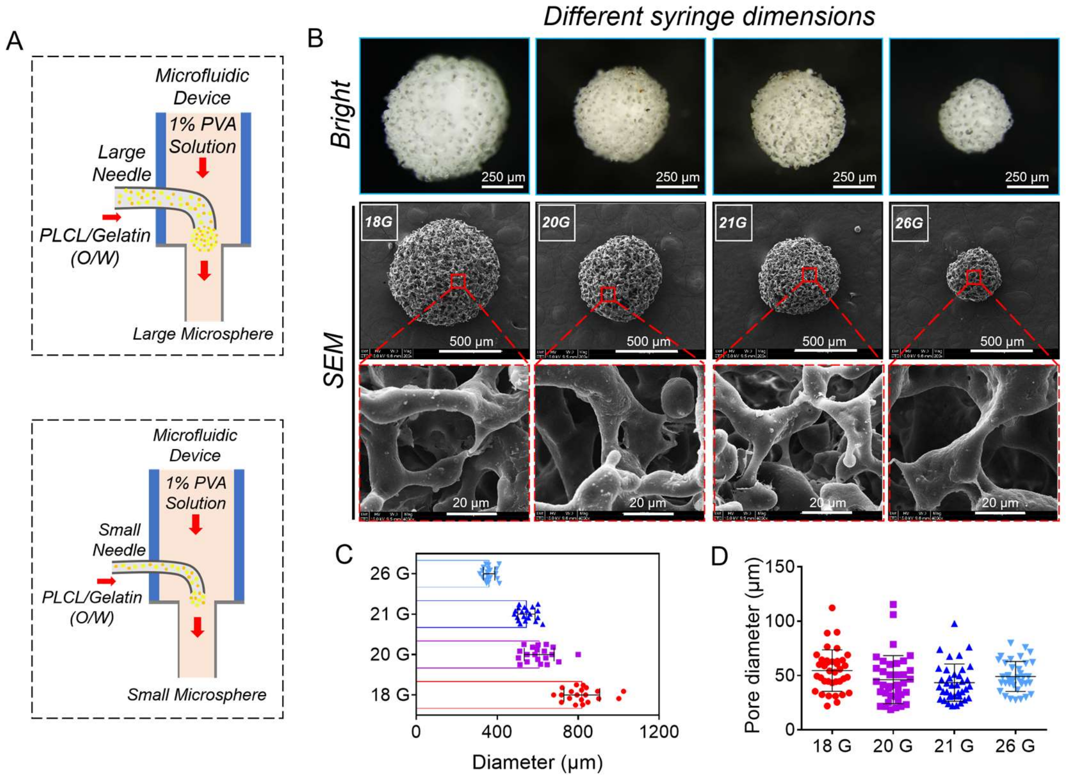


**Supplementary Fig. 1.** Preparation of porous PLCL microspheres with different needle sizes (18G, 20G, 21G and 26G). A) Schematic diagram of microfluidic device. B) Stereomicroscope and SEM characterization of porous PLCL microspheres. Red box: enlarged partial view. C) Statistical results of the diameter of microspheres. Data are presented as mean ± SD (n=20 independent microspheres, from 3 independent batches of samples). D) Statistical results of the pore size of microspheres. Data are presented as mean ± SD (n=37 independent pore sizes, from 3 independent batches of samples).

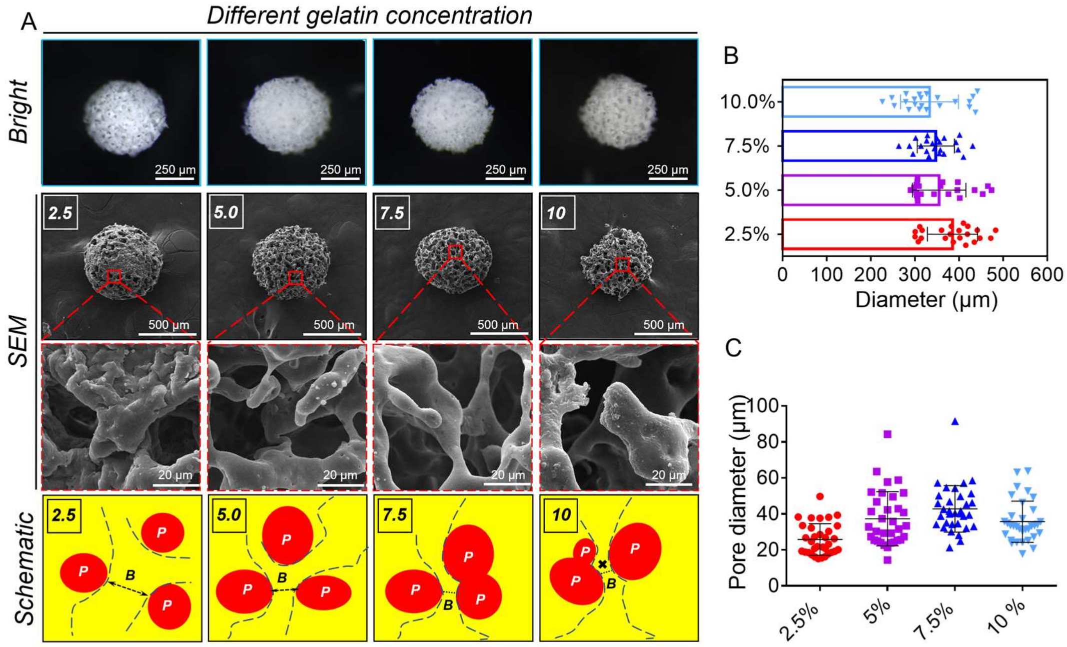


**Supplementary Fig. 2.** Preparation of porous PLCL microspheres with different gelatin concentrations (2.5%, 5.0%, 7.5% and 10.0%). A) Stereomicroscopy and SEM characterization. The schematic shows the pore distribution. Red box: enlarged partial view; P: pore structure; B: bridge. B) Statistical results of the diameter of microspheres. Data are presented as mean ± SD (n=22 independent microspheres, from 3 independent batches of samples). C) Statistical results of the pore size of microspheres. Data are presented as mean ± SD (n=33 independent pore sizes, from 3 independent batches of samples).

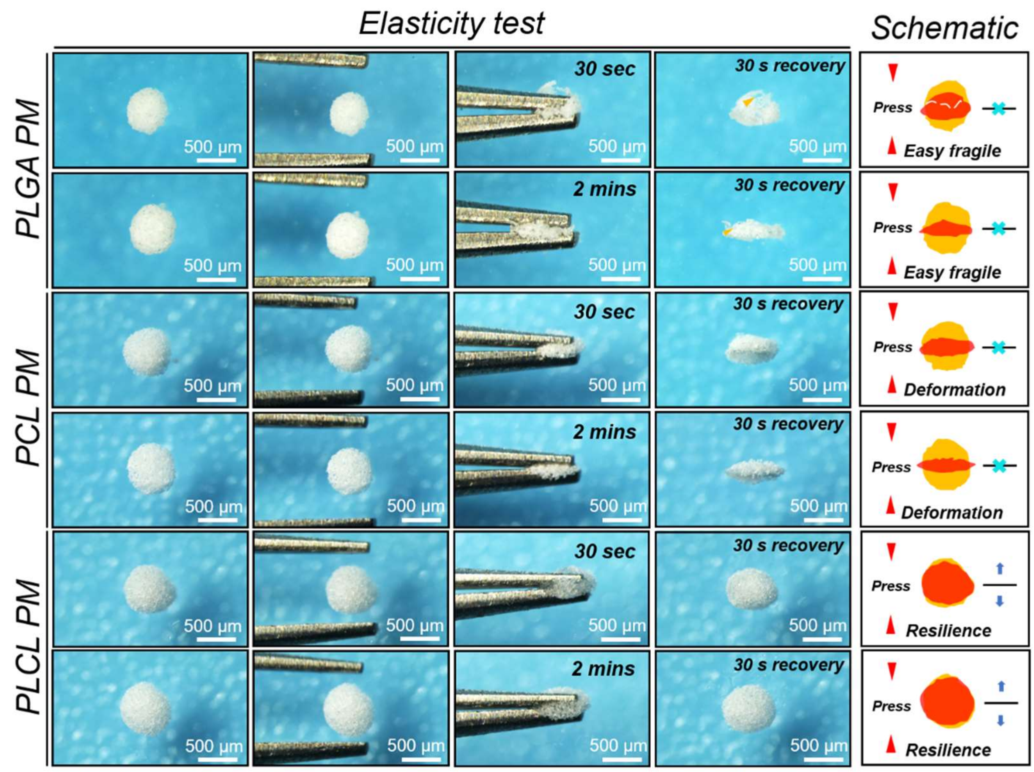


**Supplementary Fig. 3.** Physical properties characterization of porous PLCL, PLGA and PCL microspheres. The morphology of different microspheres after press a single press for 30 s and 2 mins. The schematic shows the morphological recovery of different polymeric microspheres.

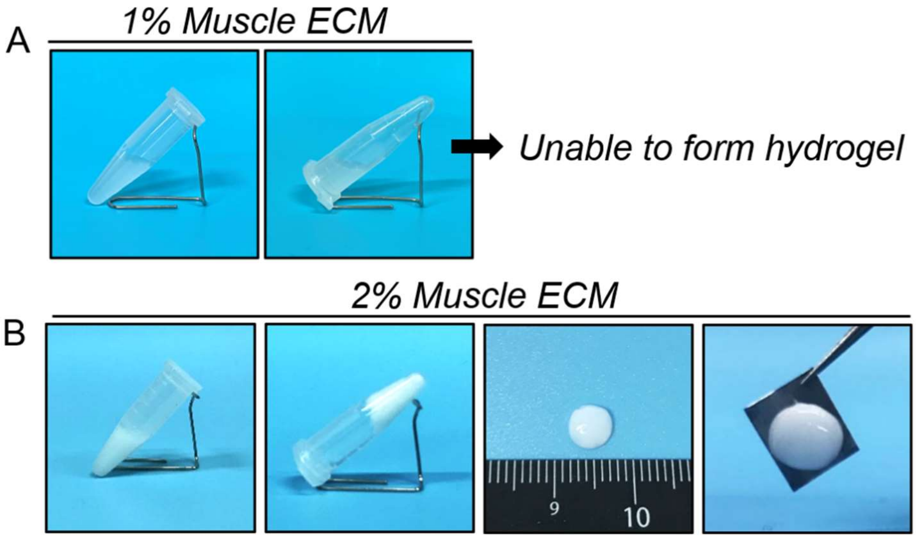


**Supplementary Fig. 4.** Characteristics of muscle ECM hydrogels with different concentrations (1% and 2%). A, B) 1% muscle ECM was not able to form a hydrogel, and the 2% muscle ECM hydrogel was unstable and could not be clamped.

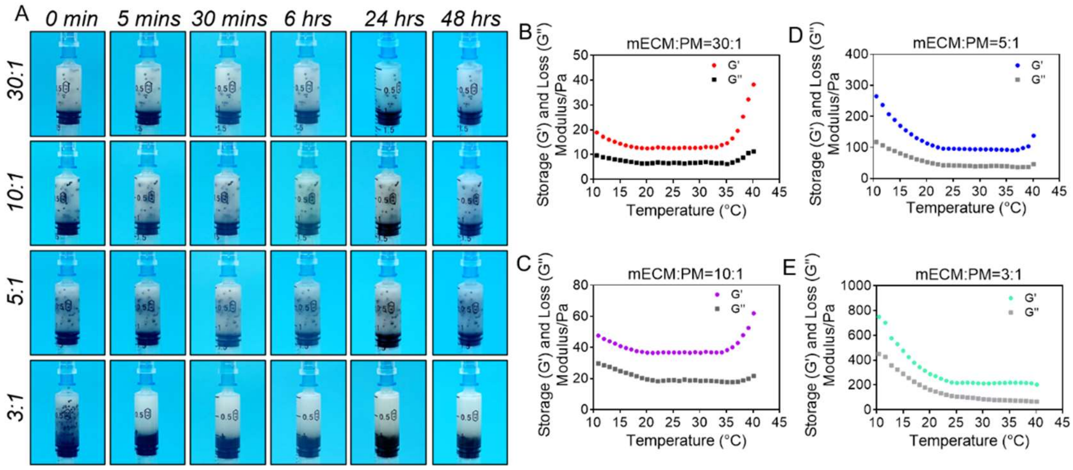


**Supplementary Fig. 5.** Stability and mechanical testing of composites with different mass ratio of mECM to PM. A) Sedimentation testing of mECM and porous microsphere composites at different time points. B-E) Rheological tests of composites.

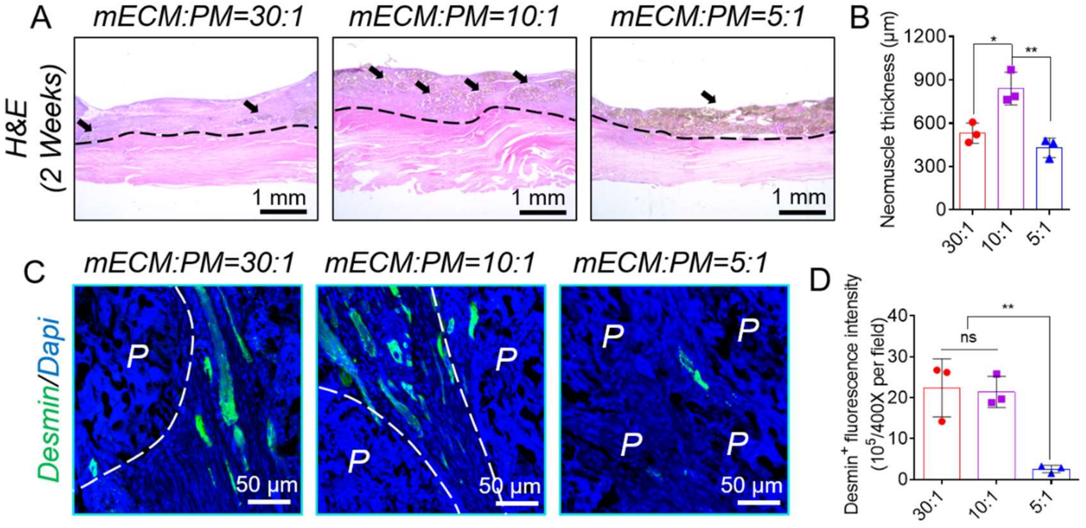


**Supplementary Fig. 6.** A) H&E staining images showing muscle regeneration. Black arrow: porous microspheres; black dashed line: the boundary between material and normal tissue. B) The statistical results of neo-muscle thickness. Data are presented as mean ± SD (n=3 biologically independent samples). $^{**}P < 0.01$, $^{*}P < 0.05$, one-way ANOVA, multiple comparisons. C) Desmin (green) immunofluorescence images showing neo-muscle regeneration. Nuclei: blue. P: porous microspheres; white dashed line: the junction between microspheres and tissues. D) Statistical analysis of desmin fluorescence intensity in C. Data are presented as mean ± SD (n=3 biologically independent samples). $^{**}P < 0.01$, ns: no significant difference, one-way ANOVA, multiple comparisons.

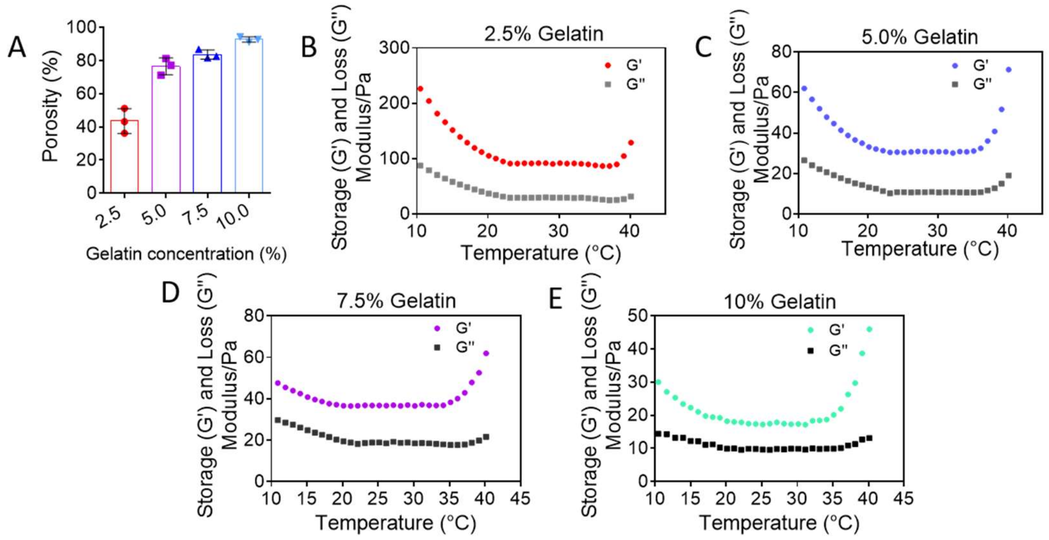


**Supplementary Fig. 7.** The effect of porosity of microspheres on the mechanical strength of composites. A) The porosity of porous PLCL microspheres at different gelatin concentrations (2.5%, 5.0%, 7.5% and 10.0%). Data are presented as mean ± SD (n=3 biologically independent samples). B-E) Rheological tests of composites containing microspheres with different porosity, and the mass ratio of mECM to PM was 10:1 in all groups.

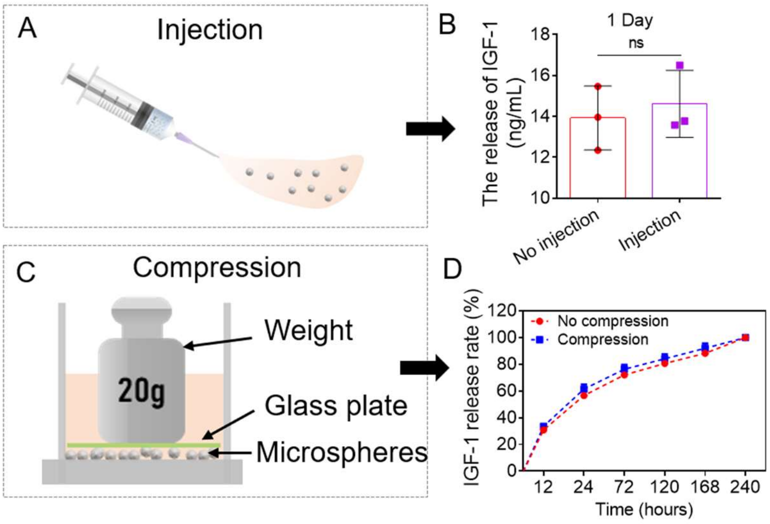


**Supplementary Fig. 8.** IGF-1 release behavior after injection and compression. A) Schematic diagram of injection in vitro. B) The release amount of IGF-1 on day 1 after injection. Data are presented as mean ± SD (n=3 biologically independent samples). ns: no significant difference, one-way ANOVA, multiple comparisons. C) Pattern diagram of parallel plate compression testing. D) Release profiles of IGF-1under compression or non-compression. Data are presented as mean ± SD (n=3 biologically independent samples).

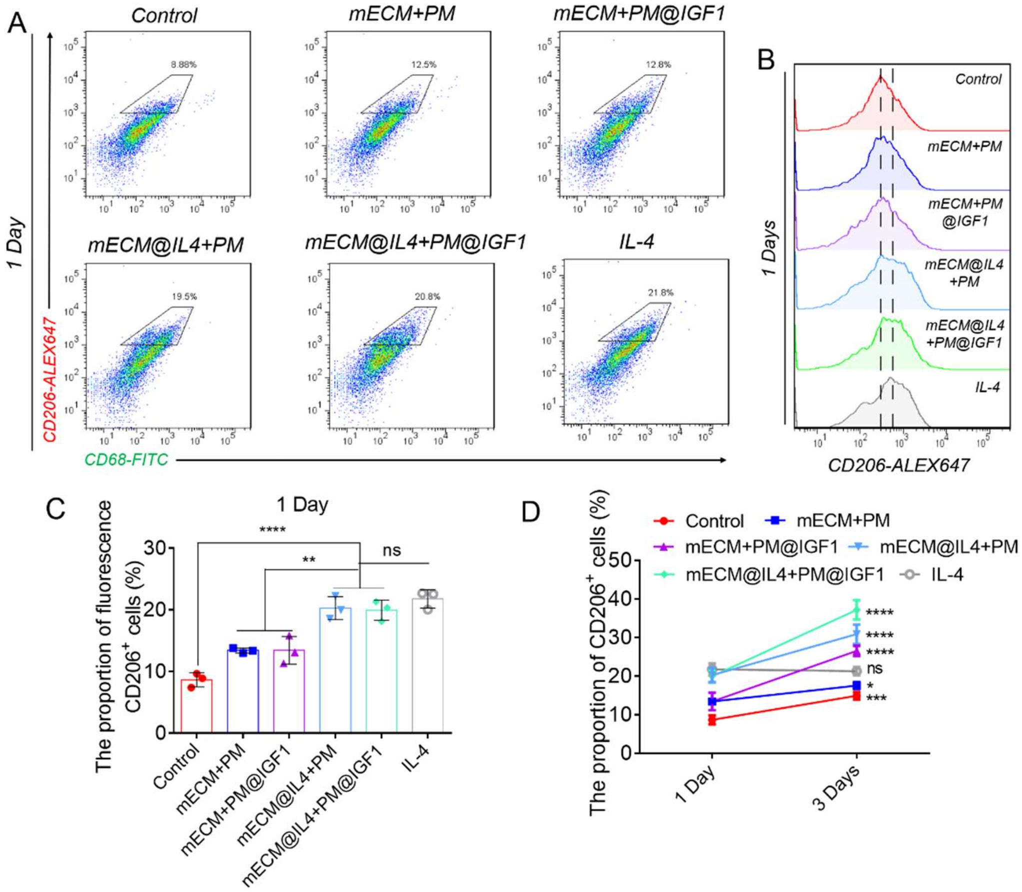


**Supplementary Fig. 9.** A) Flow cytometric results of primary macrophages treated with different composites for 1 day, mainly observing the expression of $CD206^+$ gated cells. B-C) Histogram of the peak shift of CD206 expression and corresponding statistical results. Data are presented as mean ± SD (n=3 biologically independent samples). ****$P < 0.0001$, **$P < 0.01$, ns: no significant difference, one-way ANOVA, multiple comparisons. D) The change trend of the proportion of $CD206^+$ cells from day 1 to day 3. Data are presented as mean ± SD (n=3 biologically independent samples). ****$P < 0.0001$, ***$P < 0.001$, *$P < 0.05$, ns: no significant difference, two-way ANOVA, multiple comparisons.

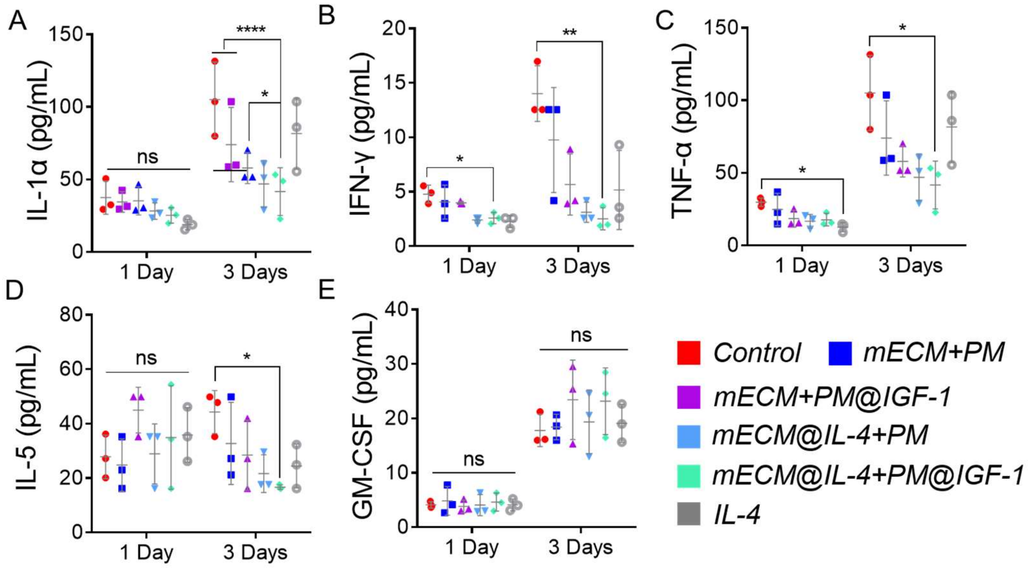


**Supplementary Fig. 10.** Luminex detected the secretion of inflammation-related factors after treatment of primary macrophages with different composites on day 1 and day 3. Data are presented as mean ± SD (n=3 biologically independent samples). ****$P < 0.0001$, **$P < 0.01$, *$P < 0.05$, ns: no significant difference. one-way ANOVA, multiple comparisons.

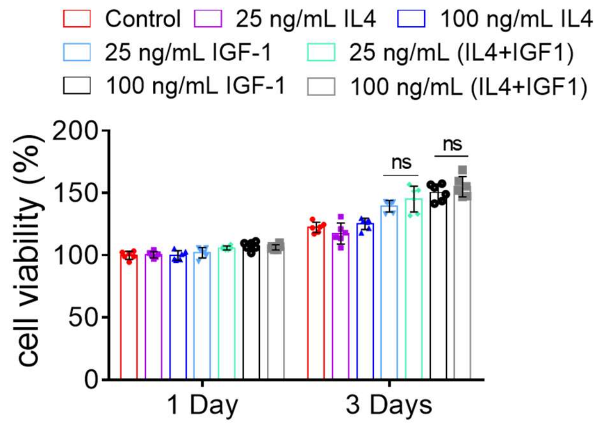


**Supplementary Fig. 11.** Cell viability of L6 cells treated with different concentration of IL-4 and IGF-1 on days 1 and 3. Data are presented as mean ± SD (n=6 biologically independent samples). ns: no significant difference, two-way ANOVA, multiple comparisons.

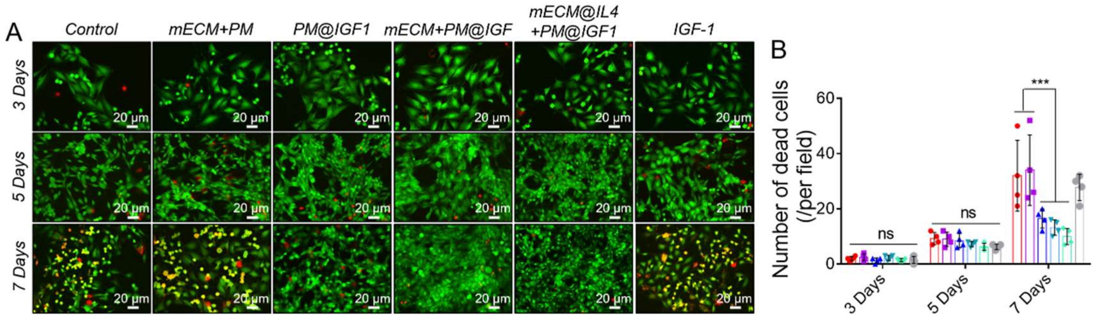


**Supplementary Fig. 12.** A) Live/Dead cell staining of L6 cells cocultured with different materials on days 3, 5 and 7. Living cells: green; dead cells: red. B) Statistical analysis of the number of dead cells on days 3, 5, and 7. Data are presented as mean ± SD (n=4 biologically independent samples). ***$P < 0.001$, ns: no significant difference, one-way ANOVA, multiple comparisons.

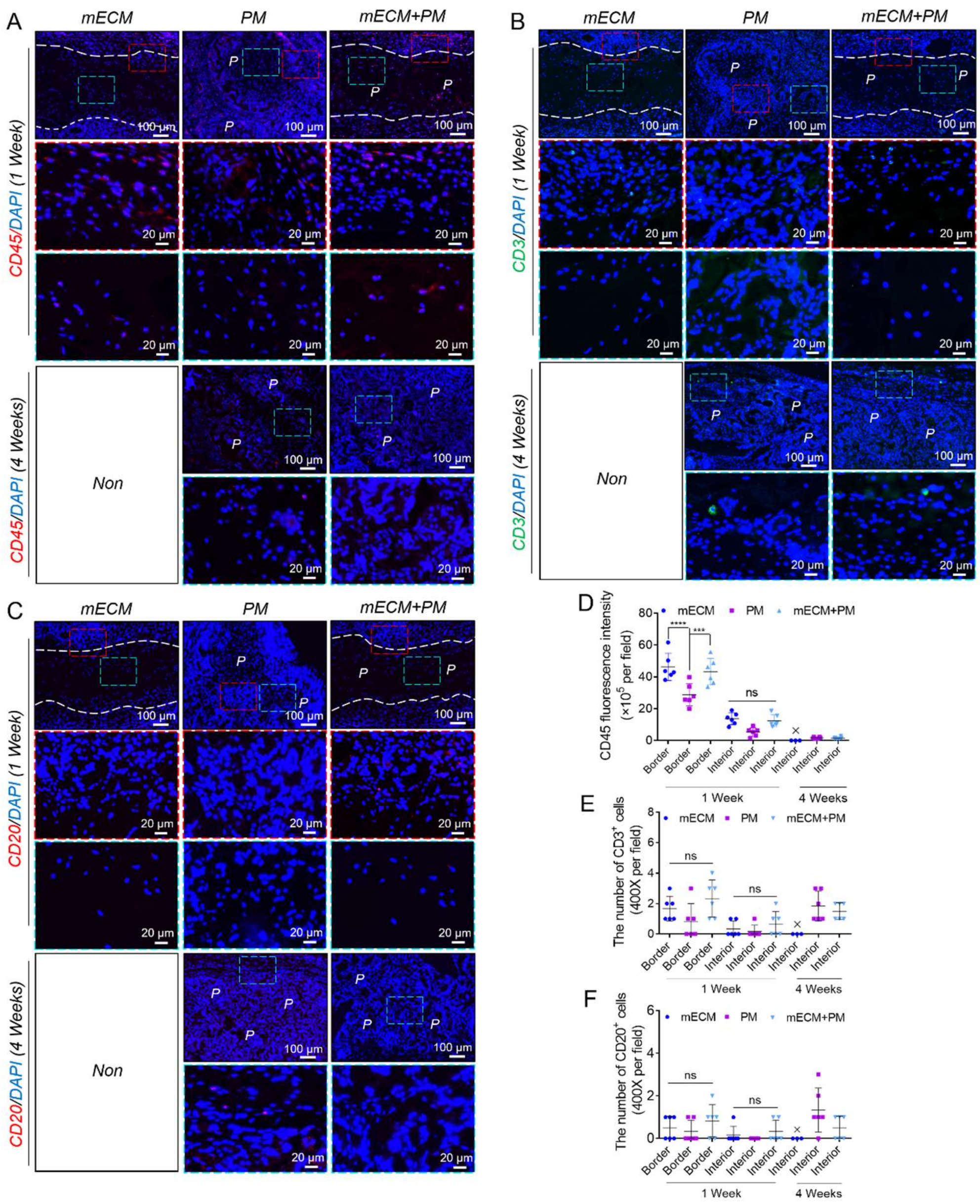


**Supplementary Fig. 13.** A-C) Immunofluorescence staining images of CD45 (red), CD3 (green) and CD20 (red) after subcutaneous injection at 1 and 4 weeks. Nuclei: blue. P: porous microspheres; light blue dotted box: central region; red dotted box: marginal region; white dashed line: the boundary between material and tissues. D-F) Statistical analysis results of CD45, CD3 and CD20. Data are presented as mean ± SD (n=3 biologically independent samples, 2 random fields per sample). ****$P < 0.0001$, ***$P < 0.001$, ns: no significant difference, one-way ANOVA, multiple comparisons.

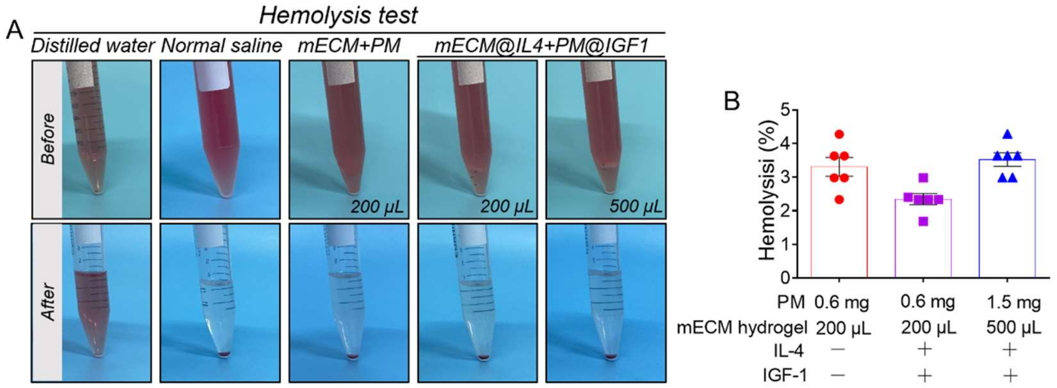


**Supplementary Fig. 14.** A) Hemocompatibility analysis of different materials. B) The statistical results of the hemolysis rate. Data are presented as mean ± SD (n=5 biologically independent samples). One-way ANOVA, multiple comparisons.

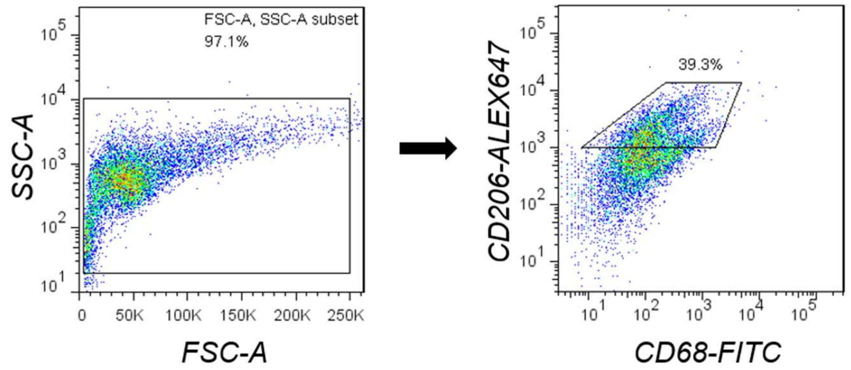


**Supplementary Fig. 15.** The gating strategy for the flow cytometry.

**Supplementary Table. 1.** Information of primary and secondary antibodies.

| Primary/secondary antibodies | Dilution Factor | Manufacturer | Cat.No |
|---|---|---|---|
| Mouse monoclonal to CD68 antibody | 1:250 | Abcam | ab31630 |
| Rabbit polyclonal to iNOS antibody | 1:200 | Abcam | ab15323 |
| Rabbit polyclonal to CD45 antibody | 1:150 | Abcam | ab10558 |
| Rabbit monoclonal to CD3 antibody | 1:100 | Abcam | ab16669 |
| Rabbit monoclonal to CD20 antibody | 1:100 | Abcam | ab64088 |
| Rabbit polyclonal to CD206 antibody | 1:300 | Abcam | ab64693 |
| Rabbit polyclonal to desmin antibody | 1:50 | Abcam | ab15200 |

| | | | |
|---|---|---|---|
| Mouse monoclonal to desmin antibody | 1:200 | Santa | sc23879 |
| Rabbit polyclonal to α-SMA antibody | 1:300 | Abcam | ab7817 |
| Mouse monoclonal to NF-09 antibody | 1:200 | Abcam | ab7794 |
| Rabbit monoclonal to TNF-α antibody | 1:100 | Abcam | ab183218 |
| FITC® 488 CD68 antibody | 1:100 | Biolegend | 137012 |
| Alexa Fluor® 647 CD206 antibody | 1:200 | Biolegend | 141712 |
| FITC phalloidin | 1:80 | Solarbio | Ca1620 |
| goat anti-rabbit IgG Alexa 594 | 1:500 | Invitrogen | A11037 |
| Goat anti-mouse IgG1 Alexa 488 | 1:500 | Invitrogen | A11029 |
| goat anti-mouse IgG Alexa 647 | 1:300 | Invitrogen | A21235 |
| goat anti- rabbit IgG Alexa 561 | 1:300 | Invitrogen | A11036 |
