# Supplementary material for: Elastic porous microspheres/extracellular matrix hydrogel injectable composites releasing dual bio-factors enable tissue regeneration
Source: Nat Commun. 2024 Feb 14;15:1377. doi: 10.1038/s41467-024-45764-4 (PMC10866888; doi:10.1038/s41467-024-45764-4)
Supplement: Supplementary file 2 — Reporting Summary [file 41467_2024_45764_MOESM2_ESM.pdf]

nature portfolio

Corresponding author(s): Meifeng Zhu

Last updated by author(s): Jan 19, 2024



# Reporting Summary

Nature Portfolio wishes to improve the reproducibility of the work that we publish. This form provides structure for consistency and transparency in reporting. For further information on Nature Portfolio policies, see our Editorial Policies and the Editorial Policy Checklist.

## Statistics

For all statistical analyses, confirm that the following items are present in the figure legend, table legend, main text, or Methods section.

| n/a | Confirmed | |
|---|---|---|
| ☐ | ☒ | The exact sample size (*n*) for each experimental group/condition, given as a discrete number and unit of measurement |
| ☐ | ☒ | A statement on whether measurements were taken from distinct samples or whether the same sample was measured repeatedly |
| ☐ | ☒ | The statistical test(s) used AND whether they are one- or two-sided<br>*Only common tests should be described solely by name; describe more complex techniques in the Methods section.* |
| ☒ | ☐ | A description of all covariates tested |
| ☒ | ☐ | A description of any assumptions or corrections, such as tests of normality and adjustment for multiple comparisons |
| ☐ | ☒ | A full description of the statistical parameters including central tendency (e.g. means) or other basic estimates (e.g. regression coefficient) AND variation (e.g. standard deviation) or associated estimates of uncertainty (e.g. confidence intervals) |
| ☒ | ☐ | For null hypothesis testing, the test statistic (e.g. *F*, *t*, *r*) with confidence intervals, effect sizes, degrees of freedom and *P* value noted<br>*Give P values as exact values whenever suitable.* |
| ☒ | ☐ | For Bayesian analysis, information on the choice of priors and Markov chain Monte Carlo settings |
| ☒ | ☐ | For hierarchical and complex designs, identification of the appropriate level for tests and full reporting of outcomes |
| ☒ | ☐ | Estimates of effect sizes (e.g. Cohen's *d*, Pearson's *r*), indicating how they were calculated |

*Our web collection on statistics for biologists contains articles on many of the points above.*

## Software and code

Policy information about availability of computer code

| | |
|---|---|
| Data collection | Stereoscopic microscope (Leica, M125-C) inverted microscope (Olympus, EP50), fluorescence microscope (Zeiss Axio Imager Z1, Germany), confocal laser microscope (Leica, SP8, Germany) and total internal reflection fluorescent microscope TIRF & Thunder (Leica, DMi8S, Germany) were used to acquire images.<br>Microplate reader (Bio-Rad, USA) was used to evaluate cell viability.<br>FACSCalibur flow cytometer (BD Biosciences) was used to acquire CD206+ macrophage cell result.<br>Magnetic bead multiplexed cytokine assays (Luminex) were used to obtain the cytokine release results.<br>Quant-iT PicoGreen Assay (Invitrogen, Grand Island, NY) was used to obtain the DNA quantitative result, α-galactosidase (α-GAL) activity detection kit (Solarbio) was used to obtain the α-GAL quantitative result, ELISA kits were used to obtain the factor release results.<br>Rheometer was used to obtain the rheology test results (G' and G").<br>UV/VIS spectrophotometer (UNIC 2802S, China) was used to obtain the hemolysis result. |
| Data analysis | Confocal image analysis was performed with a Leica SP8 microscope using LAS X.<br>Measurement and fluorescence intensity results were performed using the Image-Pro Plus 6.0 software.<br>Flow cytometric data were analyzed using FlowJo 7.6 software.<br>GraphPad Prism 6.0 software (GraphPad Software Inc., La Jolla, CA, USA) was used for statistical analysis. |

For manuscripts utilizing custom algorithms or software that are central to the research but not yet described in published literature, software must be made available to editors and reviewers. We strongly encourage code deposition in a community repository (e.g. GitHub). See the Nature Portfolio guidelines for submitting code & software for further information.

## Data

Policy information about availability of data

All manuscripts must include a data availability statement. This statement should provide the following information, where applicable:

- Accession codes, unique identifiers, or web links for publicly available datasets
- A description of any restrictions on data availability
- For clinical datasets or third party data, please ensure that the statement adheres to our policy

All data needed to support the conclusions in the study are available within the article and/or the supplementary files and movies. Data underlying Figures 1–9 and Supplementary Figures 1–13 are provided with this paper in the source data file. Any additional requests for information can be directed to, and will be fulfilled by the corresponding authors. Source data files are provided with this paper.

## Research involving human participants, their data, or biological material

Policy information about studies with human participants or human data. See also policy information about sex, gender (identity/presentation), and sexual orientation and race, ethnicity and racism.

| | |
|---|---|
| Reporting on sex and gender | we don't have related issues. |
| Reporting on race, ethnicity, or other socially relevant groupings | we don't have related issues. |
| Population characteristics | we don't have related issues. |
| Recruitment | we don't have related issues. |
| Ethics oversight | we don't have related issues. |

Note that full information on the approval of the study protocol must also be provided in the manuscript.

# Field-specific reporting

Please select the one below that is the best fit for your research. If you are not sure, read the appropriate sections before making your selection.

[x] Life sciences [ ] Behavioural & social sciences [ ] Ecological, evolutionary & environmental sciences

For a reference copy of the document with all sections, see nature.com/documents/nr-reporting-summary-flat.pdf

# Life sciences study design

All studies must disclose on these points even when the disclosure is negative.

| | |
|---|---|
| Sample size | The pore size of mECM and porous microspheres was calculated through the image pro-puls software. The diameter of porous microspheres was measured using the stereoscopic microscope(Leica, M125-C), the measurement results were directly recorded in the experimental notebook, without taking photos of all the recorded porous microspheres. The recorded results were consistent with the description in article. |
| Data exclusions | No data were excluded from the analysis. |
| Replication | Performance testing of porous microspheres were done at least three times, but in Fig.2 (repeated compressions detection), we only counted the continuous morphology recovery of individual microspheres.<br>All light microscopy experiments were done in triplicate.<br>All experiments were repeated from at least three independent tests, and all attempts at replication were successful. |
| Randomization | Materials (eg.microspheres) and tissue sections used for imaging were selected randomly, and SD rats for animal experiments were also divide into groups randomly. |
| Blinding | The persons performing statistical analysis were unaware of the sample identity. |

# Reporting for specific materials, systems and methods

We require information from authors about some types of materials, experimental systems and methods used in many studies. Here, indicate whether each material, system or method listed is relevant to your study. If you are not sure if a list item applies to your research, read the appropriate section before selecting a response.

## Materials & experimental systems

| n/a | Involved in the study |
|---|---|
| ☐ | ☒ Antibodies |
| ☐ | ☒ Eukaryotic cell lines |
| ☒ | ☐ Palaeontology and archaeology |
| ☐ | ☒ Animals and other organisms |
| ☒ | ☐ Clinical data |
| ☒ | ☐ Dual use research of concern |
| ☒ | ☐ Plants |

## Methods

| n/a | Involved in the study |
|---|---|
| ☒ | ☐ ChIP-seq |
| ☐ | ☒ Flow cytometry |
| ☒ | ☐ MRI-based neuroimaging |

# Antibodies

| | |
|---|---|
| Antibodies used | CD68 mouse monoclonal antibody(1:250, ab31630, Abcam)<br>iNOS rabbit polyclonal antibody (1:300,ab15323,Abcam)<br>CD206 rabbit polyclonal antibody(1:300,ab64693,Abcam)<br>TNF-α rabbit monoclonal antibody (1:100, ab183218,Abcam)<br>α-SMA rabbit polyclonal antibody ( 1: 300, ab7817,Abcam)<br>Desmin rabbit polyclonal antibody (1:50, ab15200 Abcam)<br>Desmin mouse monoclonal antibody (1:200, sc23879, Santa)<br>NF-09 Mouse monoclonal antibody (1:200, ab7794,Abcam)<br>CD45 (Rabbit polyclonal antibody (1:150, ab10558, Abcam)<br>CD20 rabbit monoclonal antibody (1:100, ab64088, Abcam)<br>CD3 rabbit monoclonal antibody(1:100, ab16669, Abcam)<br>Phalloidin (1:80, ca1620, Solarbio)<br>goat anti-rabbit lgG Alexa 594 (1:500,a11037,Invitrogen,USA)<br>goat anti-mouse IgG1 Alexa 488 (1:500,a11029,Invitrogen, USA)<br>goat anti-mouse lgG Alexa 647(1:300,a21235,Invitrogen,USA)<br>goat anti-rabbit lgG Alexa 568 (1:300,a11036, Invitrogen,USA)<br>FITC-488 CD68 antibody (1:100,137012, Biolegend)<br>Alexa Fluor-647 CD206 antibody (1:200, 141712,Biolegend) |
| Validation | Desmin and NF-09 antibodies were validated in our previous work (Deling Kong and Meifeng Zhu, 2019).<br>CD68, iNOS, CD206 and α-SMA antibodies were also validated in our previous work (Meifeng Zhu and Siyang Liu, 2022).<br>Other antibodies were first used in this research and exhibited great results .All antibodies can be queried on the corresponding official website. |

# Eukaryotic cell lines

Policy information about cell lines and Sex and Gender in Research

| | |
|---|---|
| Cell line source(s) | L6 cells (Rat, ATCC, CRL-1458) |
| Authentication | None of the cell lines have been authenticated. |
| Mycoplasma contamination | Cell lines were not tested for mycoplasma contamination but no indication of contamination was observed. |
| Commonly misidentified lines<br>(See ICLAC register) | No commonly misidentified cell lines were used. |

# Animals and other research organisms

Policy information about studies involving animals; ARRIVE guidelines recommended for reporting animal research, and Sex and Gender in Research

| | |
|---|---|
| Laboratory animals | 75 male sprague-Dawley rats aged 8-10 weeks with the weight range of 280-300g.<br>30 male sprague-Dawley rats aged 4-5 weeks with the weight range of 120-150g.<br>42 male sprague Dawley mammary rats ( 5 days) were used in this study. |
| Wild animals | No wild animals were used in this study. |
| Reporting on sex | we don't have related issues. |
| Field-collected samples | No field-collected samples were used in this study. |
| Ethics oversight | All animal experiments were approved by the animal experiments ethical committee of Nankai University, Tianjin, China (2022- |

SYDWLL-000432) and met the requirements of the Guidelines for Care and Use of Laboratory Animals.
Rats were randomly divided into cages (3 rats per cage) and adapted to a pellet-based diet with tap water for 7 days in an SPF environment. The ambient temperature was maintained at 23 ± 3℃, humidity at 40%-70%, and cycle light/dark for 12 hrs. Rats were fed a standard food diet and tap water. And during the feeding period, the animal room was cleaned regularly. Rats were fasting 12 hrs pre-surgery but can have water. After surgery, all experimental animals were lying sideways on a heating blanket (37 ℃) to maintain their smooth breathing and body temperature within 2 hrs. Paid attention to maintaining a quiet environment until the animals were completely awake. All experimental animals were fasting 12 hrs post-surgery but can drink freely. During the wound recovery period, we also paid attention to animals' physical conditions, such as feeding, excretion, surgical infection, and suture status. In addition, we recorded the local reaction of the injection site after injecting different materials in the rat subcutaneous injection model and VML model, including visible swelling, redness, edema, and abnormal color.

Note that full information on the approval of the study protocol must also be provided in the manuscript.

# Plants

Seed stocks

we don't have related issues.

Novel plant genotypes

we don't have related issues.

Authentication

we don't have related issues.

# Flow Cytometry

## Plots

Confirm that:

- [x] The axis labels state the marker and fluorochrome used (e.g. CD4-FITC).
- [x] The axis scales are clearly visible. Include numbers along axes only for bottom left plot of group (a 'group' is an analysis of identical markers).
- [x] All plots are contour plots with outliers or pseudocolor plots.
- [x] A numerical value for number of cells or percentage (with statistics) is provided.

## Methodology

| | |
|---|---|
| Sample preparation | Detailed information about the flow cytometry experiments are provided in 'materials and methods' section. |
| Instrument | FACSCalibur flow cytometer (BD Biosciences). |
| Software | CellQuest software (Pharminogen) and FlowJo software. |
| Cell population abundance | Before flow cytometry testing, we purified cells to reduce interference from other cells. And during the testing process, we first adjust the FSC and SSC to distribute the target cells in the appropriate position on the X&Y axis, and excluded interference from cell fragments (FSC-low regions). Subsequently, 10000 cells were collected for subsequent analysis, CD68+/ CD206- cells were used as negative group. |
| Gating strategy | We set CD68+/CD206- cells as the negative group, and mainly observed the proportion of CD68+/CD206+ cells in every groups. |

- [x] Tick this box to confirm that a figure exemplifying the gating strategy is provided in the Supplementary Information.
