# Supplementary material for: Elastic porous microspheres/extracellular matrix hydrogel injectable composites releasing dual bio-factors enable tissue regeneration
Source: Nat Commun. 2024 Feb 14;15:1377. doi: 10.1038/s41467-024-45764-4 (PMC10866888; doi:10.1038/s41467-024-45764-4)
Supplement: Supplementary file 3 — Description of Additional Supplementary Files [file 41467_2024_45764_MOESM3_ESM.pdf]

Title: Supplementary movie 1:

Description: Movie of pressing of porous PLCL microspheres.

Title: Supplementary movie 2:

Description: Movie of pressing of porous PLGA microspheres.

Title: Supplementary movie 3:

Description: Movie of pressing of porous PCL microspheres.

Title: Supplementary movie 4:

Description: Movie of injectability of composites with the mass ratio of mECM to PM 30:1.

Title: Supplementary movie 5:

Description: Movie of injectability of composites with the mass ratio of mECM to PM 10:1.

Title: Supplementary movie 6:

Description: Movie of injectability of composites with the mass ratio of mECM to PM 5:1.

Title: Supplementary movie 7:

Description: Movie of injectability of composites with the mass ratio of mECM to PM 3:1.

Title: Supplementary movie 8:

Description: Movie of subcutaneous injection of porous PLCL microspheres (PM group) in rat.

Title: Supplementary movie 9:

Description: Movie of subcutaneous injection of mECM hydrogels in rat.

Title: Supplementary movie 10:

Description: Movie of subcutaneous injection of mECM+PM composites in rat.
